# Is the Current Management of Severe Sepsis and Septic Shock Really Evidence Based?

**DOI:** 10.1371/journal.pmed.0030346

**Published:** 2006-08-22

**Authors:** Jean-Louis Vincent

## Abstract

Vincent discusses the reasons why evidence-based medicine may be particularly difficult to apply in the management of patients with severe sepsis.

Evidence-based medicine (EBM), a process of accurately assessing and integrating the weight carried by various levels of available evidence, (e.g., randomized controlled trials [RCTs], case-control studies, case series, and expert opinion) has become the cornerstone of good medical practice. The RCT is considered the “gold-standard” of EBM, but, while the RCT may indeed be the most statistically unbiased method of assessing an intervention, it can also have limitations and must be correctly interpreted, aided by other forms of evidence.

In addition, in certain fields of medicine, such as management of patients with severe sepsis, this “high-grade” RCT evidence is in short supply [1]. The heterogeneous nature of the intensive-care-unit (ICU) population and the complexity of the disease processes involved make it difficult to show the impact of acute interventions on long-term outcomes in this setting. In addition, many treatment strategies are life saving (such as mechanical ventilation in respiratory failure, blood transfusions in acute hemorrhage, and administration of vasopressor agents in severe shock), and could not ethically be studied in a placebo-controlled RCT [1].

In view of these uncertainties, it is difficult to provide guidelines for the management of the patient with sepsis. Yet severe sepsis is a serious health-care problem, affecting some 30% of ICU admissions and associated with ICU mortality rates of around 30%; septic shock is associated with ICU mortality rates of around 50%. There is no doubt that better outcomes could be achieved in this patient population.

## Current Guidelines

The American College of Critical Care Medicine recently developed guidelines for the management of the hemodynamically unstable patient with sepsis [2]. More general guidelines were produced by the Surviving Sepsis Campaign by using a modified Delphi methodology with a group of about 50 international critical care and infectious disease experts [3]. All aspects of management of the patient with severe sepsis were covered (Box 1), and recommendations, graded from A to E depending on the level of evidence available, were developed for each category. However, these guidelines and their application have generated much debate. A closer look at the Surviving Sepsis Campaign guidelines shows the low level of confidence that the authors have in the evidence behind the interventions: most interventions were only given an E grade (Figure 1).

**Figure 1:**
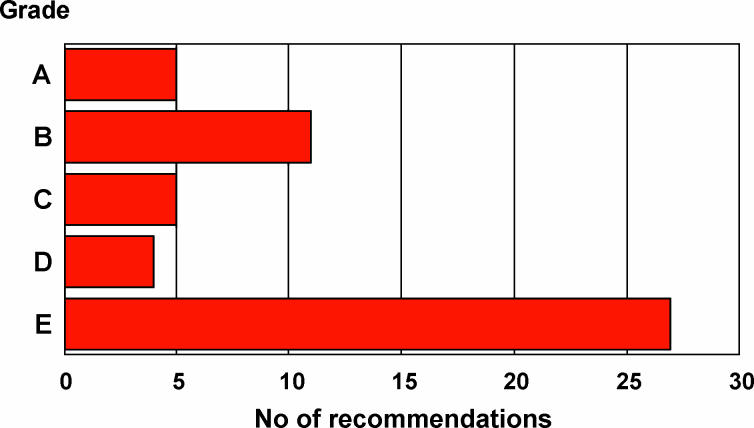
Number of Recommendations of Each Grade of Evidence in the Surviving Sepsis Campaign Guidelines The figure shows that there are more E-grade recommendations than all the other grades put together. Grading system: A, supported by at least two large randomized trials with clear results; B, supported by a large randomized trial with clear results; C, supported by small randomized trial(s) with uncertain results; D, supported by a study with nonrandomized contemporaneous controls; E, studies with historical controls, uncontrolled studies, case series, and expert opinion.

**Box 1. Aspects of Patient Management Covered by the Surviving Sepsis Campaign Guidelines**

- Initial resuscitation
- Diagnosis
- Antibiotic therapy
- Source control
- Fluid therapy
- Vasopressors
- Inotropic therapy
- Steroids
- Activated protein C
- Blood product administration
- Mechanical ventilation
- Sedation/analgesia
- Glucose control
- Renal replacement
- Bicarbonate therapy
- Deep-vein thrombosis prophylaxis
- Stress-ulcer prophylaxis
- Consideration for limitation of life support

(Data obtained from [3])

## Limitations to Guidelines for the Management of the Patient with Sepsis

There are many reasons why EBM may be particularly difficult to apply, and hence result in somewhat limited guidelines, in the management of patients with severe sepsis. I have selected just three possible reasons below, drawing examples from the Surviving Sepsis Campaign guidelines to illustrate each point.

## No RCT Evidence Available

There are many areas in the management of the patient with severe sepsis that have never been subjected to RCTs, and recommendations must be based on other grades of evidence. Here I give just two examples.

### Antibiotic therapy

The beneficial effects of antibiotic therapy, per se, have never been specifically tested in an RCT, although RCTs have been conducted that compare different antibiotics in various specific infections. The only recommendation that is supported by clinical studies is that antibiotics that cover all likely organisms should be prescribed [4–7]. It also seems logical that if antimicrobial therapies are effective, they should be started as early as possible. However, it is difficult to administer agents that can cover all possible organisms in every case; should we, for example, treat a patient who has pneumonia that is likely a result of *Pneumococcus* with broad-spectrum antibiotics? In addition, should empiric antibiotics be administered to all patients with suspected infection, so that patients who are infected have the benefit of receiving early therapy, or should we wait until infection is proven in order to limit the negative effects and costs of unnecessary antimicrobial treatment?

### Vasoactive agents

Many patients with septic shock will need vasopressor agents, but which agent is best? Dopamine has inotropic properties in addition to its vasopressor effects, thus creating a sort of “inoconstricting” agent, and its dopaminergic effects may preferentially direct blood flow to the kidneys and the gut, although admittedly, this does not prevent renal failure [8]. On the other hand, norepinephrine may have beneficial effects on renal function during sepsis [9]. Epinephrine is also used routinely in some centers. No RCT has directly compared these agents in patients with severe sepsis. A multicenter French study evaluating the effects of epinephrine versus the effects of a combination of norepinephrine with dobutamine has been completed recently; there was no difference in mortality in the two groups (D. Annane, unpublished data). A multicenter RCT comparing norepinephrine with dopamine as first-line vasopressors in septic shock is currently underway in Europe.

## RCT Evidence Available, but Not Specifically in Patients with Sepsis

There are interventions that may be relevant to patients with sepsis and that have undergone testing in RCTs, but these RCTs were not conducted specifically in patients with sepsis. Instead, they were conducted in general populations of ICU patients, some of whom may have had a diagnosis of sepsis. Extrapolation of these results in general, or from subgroup analyses, can lead to recommendations, but are such recommendations really relevant to the patient with sepsis?

### Fluid administration

The optimal fluid for resuscitating the patient with septic shock is a controversial topic. Although there is no evidence that colloids are any better than crystalloids, there is a strong rationale to attempt to limit the formation of edema. The large Saline versus Albumin Fluid Evaluation (SAFE) study in Australasia, which compared albumin and saline in patients in intensive care, showed similar outcomes with these two different fluids at 28 days [10]. But in the subgroup of patients with severe sepsis, 185 (30.7%) of the 603 patients with severe sepsis who had been assigned to receive albumin died, and 217 (35.3%) of the 615 patients with severe sepsis who had been assigned to receive saline died (relative risk, 0.87; 95% confidence interval, 0.74–1.02; *p* = 0.09). Hence, there was a suggestion that albumin may be associated with improved outcomes in this population of patients, but subgroup analyses carry many limitations, including reduced statistical power, and reliance on such results may be erroneous [11]. Further studies are necessary that specifically target patients with severe sepsis and septic shock, and that take the severity of hypoalbuminemia into account.

### Tight blood sugar control

Van den Berghe et al. [12] showed that patients in the surgical ICU who received intensive insulin therapy (to maintain blood glucose levels at 80–110 mg/dl) had reduced mortality compared with those who were managed conventionally (infusion of insulin only if blood glucose levels exceeded 215 mg/dl). The reduction was greatest for deaths caused by multiple-organ failure with a proven septic focus. Although compelling and potentially important for patients with sepsis, these observations were made in a single institution, and were not specifically targeted at patients with sepsis. In addition, maintaining tight blood sugar control can be a challenge for staff, especially since the risk of hypoglycemia is of great concern in unstable patients with sepsis. A recent multicenter study in Germany was discontinued prematurely because of identical mortality rates in the treatment groups and a greater incidence of hypoglycemia in the tight blood sugar group (K. Reinhart, unpublished data).

### Blood transfusions

In 838 critically ill patients, Hebert et al. [13] randomized patients to receive a restrictive transfusion strategy, maintaining hemoglobin levels at 7–9 g/dl, or a more liberal approach with hemoglobin levels maintained at 10–12 g/dl. The restrictive approach was at least as effective as the liberal approach and perhaps more so in younger and less severely ill patients. However, this study again included a general population of critically ill patients and was not focused specifically on patients with sepsis. In addition, although the observational Anemia and Blood Transfusion in the Critically Ill (ABC) study [14] supported the suggestion that higher transfusion rates were associated with increased mortality, the recent Sepsis Occurrence in Acutely Ill Patients (SOAP) study [15] found no association between transfusion and mortality. Moreover, Rivers et al. [16] showed that patients in an early goal-directed therapy group, with increased survival rates, were more likely to have received transfusions.

How do we account for these apparent differences? Perhaps they are related to a change in the quality of transfusion since the original Hebert study [13], for which the first data were collected almost ten years ago. Indeed, since that study, deleukocytation of blood is now routine practice in many countries and leukoreduced blood has been shown to be associated with reduced mortality [17]. A large study by the SOAP collaboration is under way in Europe to reevaluate transfusion thresholds in a heterogeneous group of critically ill patients.

## RCT Results Available, but Questions Remain

### Mechanical ventilation

An important study conducted by the Acute Respiratory Distress Syndrome (ARDS) Network [18] in 861 patients with acute lung injury (ALI) or ARDS was stopped at interim analysis, as patients who were randomized to mechanical ventilation with “low” tidal volumes of 6 ml/kg had a lower mortality rate than those ventilated with tidal volumes of 12 ml/kg. At face value, therefore, this study provides strong evidence that patients with ALI and ARDS should be ventilated with low tidal volumes. However, only two tidal volumes were compared and the high tidal volume chosen for the study was somewhat higher than the values usually applied [19]. Hence, the results of this study do not necessarily mean that all patients should be ventilated with tidal volumes of 6 ml/kg, but rather that ventilating patients at volumes of 12 ml/kg is detrimental. In addition, whether these results apply to all patients with severe sepsis, even when they do not meet the criteria for ALI/ARDS, has not yet been established.

### Steroids

Steroid administration in septic shock has been a matter of debate for several decades. The administration of large boluses of methylprednisolone was not shown to be beneficial in large controlled studies [20,21]. More recently, the concept of relative adrenal insufficiency has led to the consideration of administering moderate doses of steroids (200–300 mg/day of hydrocortisone) in septic shock. But a multicenter RCT [22] comparing moderate dose steroids with placebo failed to show significant differences in survival rates in the initial analysis. Complex statistical calculation was needed to show only a trend for improved outcome in the global population, although the differences became statistically significant in patients with an abnormal response to a 250-μg cosyntropin test.

Nevertheless, other recent studies have suggested improved outcomes in patients with septic shock who received moderate doses of hydrocortisone, so that its use is recommended in the Surviving Sepsis guidelines [3]. But should all patients with septic shock receive this regimen? Some experts use a 250-μg cosyntropin test to identify patients who could benefit more from steroid administration [22]; others suggest that single cortisol measurements may be sufficient [23]. And still others have proposed that a 1-μg test may be more physiological [24], and some suggest that no measurements can be interpreted adequately. So, there is clearly no consensus on this issue.

### Drotrecogin alfa (activated)

Despite many years of research, only one agent—drotrecogin alfa (activated)—has been identified that specifically affects the sepsis response and by doing so improves survival [25]. The efficacy of the drug has been shown in a large RCT, the Recombinant Human Activated Protein C Worldwide Evaluation in Severe Sepsis (PROWESS) study [25], and has been supported by the open-label Extended Evaluation of Recombinant Human Protein C (ENHANCE) study [26]. Yet, despite the RCT evidence, some people still challenge the efficacy of the drug [27], fueled further by the negative results of the recent Administration of Drotrecogin alfa (activated) in Early Stage Severe Sepsis (ADDRESS) study [28] conducted in patients with severe sepsis but with a low risk of death. Drotrecogin alfa (activated) is therefore licensed for adult patients at “high risk of death,” and one of the problems is how best to categorize such patients.

The Food and Drug Administration suggests that the APACHE II score (http://www.sfar.org/scores2/apache22.html) may be used, with treatment restricted to patients with an APACHE II score greater than 25. But this may not be the most reliable method for choosing patients for several reasons, including the following: (1) the use of drotrecogin alfa (activated) has not been validated in this context; (2) there is considerable variability in the calculation of this score [29,30]; and (3) age is included in the APACHE II score, making it more likely that elderly patients meet the criteria for administration than younger patients. Criteria for choosing patients that are based on organ failure may be more effective, but further study is necessary to optimize the use of this drug. The results of data analysis from an integrated database (INDEPTH) of placebo and drotrecogin alfa–treated patients from five severe sepsis trials have recently been published that support a survival benefit associated with drotrecogin alfa (activated) treatment compared with placebo [31].

## Conclusion

Many uncertainties remain within the field of intensive-care medicine, and in sepsis in particular. EBM has been promoted as the way to practice medicine, but in some patient groups—for example, patients with sepsis—EBM can be difficult to apply. Recently developed guidelines reflect this problem with very few recommendations being supported by the highest level of evidence. The results of ongoing and future trials will expand the evidence base, and current guidelines need to be adapted accordingly to ensure that patients continue to be treated with the very latest and best standard of care.

## Abbreviations

ALI: acute lung injury
ARDS: acute respiratory distress syndrome
EBM: evidence-based medicine
ICU: intensive care unit
RCT: randomized controlled trial
SOAP: Sepsis Occurrence in Acutely Ill Patients
