# Correction: Is the Current Management of Severe Sepsis and Septic Shock Really Evidence Based?

**DOI:** 10.1371/journal.pmed.0030543

**Published:** 2006-12-26

**Authors:** Jean-Louis Vincent

In *PLoS Medicine*, volume 3, issue 9: doi:10.1371/journal.pmed.0030346

The competing interests statement was incomplete and should have read: “Jean-Louis Vincent is the current chairman of the International Sepsis Forum, and a member of the steering committee of the Surviving Sepsis Campaign. His personal views do not necessarily reflect those of the other members of these committees. The Surviving Sepsis Campaign has received funding from Eli Lilly, Baxter, and Edwards. The International Sepsis Forum is currently sponsored by bioMérieux, Biosite, Brahms Diagnostics, EBI, Eli Lilly Corporation, Eisai Global Clinical Development, GlaxoSmithKline, Novo Nordisk, Roche Diagnostics, Spectral Diagnostics, Takeda, and Toray. The author declares that he has received grants and/or honorariums from AstraZeneca, Baxter, bioMérieux, Biosite, Brahms Diagnostics, Edwards LifeSciences, Eli Lilly Corporation, Eisai Global Clinical Development, GlaxoSmithKline, Novo Nordisk, Spectral Diagnostics, Takeda, Toray, and Wyeth.”

